# Functional impairment in a dementia‐free sample associated with a higher risk of Mild Behavioral Impairment

**DOI:** 10.1002/alz.088099

**Published:** 2025-01-03

**Authors:** Moyra E Mortby, Zahinoor Ismail, Kaarin J. Anstey

**Affiliations:** ^1^ University of New South Wales, Sydney, NSW Australia; ^2^ Hotchkiss Brain Institute, University of Calgary, Calgary, AB Canada

## Abstract

**Background:**

Mild Behavioral Impairment (MBI) and functional ability are two non‐cognitive markers of dementia. To date, little is known about the impact of functional ability on the clinical manifestation of MBI. Using data from the Australian population‐based PATH Through Life Study we examined the impact of functional ability on MBI. We hypothesized that poorer functional ability in a dementia‐free sample (with at most Mild Cognitive Impairment; MCI) would be associated with a higher likelihood of MBI.

**Method:**

1377 dementia‐free participants (52% male; aged 72‐79; 847 cognitively healthy, 397 cognitively normal, but‐at‐risk (i.e., subjective cognitive decline), 133 MCI) were included. Functional ability was assessed using the Bayer Activities of Daily Living scale (B‐ADL), which comprises 25 items assessed by an informant. Items are scored 1‐10 with higher scores indicating poorer functional ability. Presence and absence of MBI and its five domains (decreased motivation, affect dysregulation, impulse dyscontrol, social inappropriateness, abnormal perception/thought content) were approximated using a previously published transformation algorithm, which utilizes items from the Neuropsychiatric Inventory assessed at a single study visit. Binomial logistic regression analyses were conducted to examine risk of MBI associated with functional impairment. Models controlled for Mini Mental State Exam scores (MMSE) and self‐reported gender (male/female).

**Result:**

Adjusted logistic regression (MMSE and gender) showed that a one‐unit increase on the B‐ADL was associated with a 2.27‐fold greater risk of MBI (95% CI 1.962‐2.624; p<.001). Similarly, for all 5 MBI domains, a one‐unit increase of B‐ADL was associated with greater risk of all domains: decreased motivation (B = 2.26; 95% CI 1.898‐2.684; p<.001), affect dysregulation (B = 2.02; 95% CI 1.763‐2.306; p<.001), impulse dyscontrol (B = 2.01; 95% CI 1.756‐2.301; p<.001), social inappropriateness (B = 2.01; 95% CI 1.707‐2.378; p<.001), and abnormal perception/thought content (B = 1.82; 95% CI 1.417‐2.335; p<.001).

**Conclusion:**

This study is amongst the first to identify a higher risk of MBI associated with functional ability in dementia‐free older adults. These findings highlight the importance of early monitoring of changes in functional ability and behaviour, even prior to the onset of cognitive symptomatology. They also provide further evidence of the clinical utility of functional ability and MBI as early markers of dementia.

